# Association of neutrophil to lymphocyte ratio (NLR) with angiographic SYNTAX score in patients with non-ST-Segment elevation acute coronary syndrome (NSTE-ACS)

**DOI:** 10.34172/jcvtr.2021.40

**Published:** 2021-08-25

**Authors:** Mehdi Maleki, Arezou Tajlil, Ahmad Separham, Bahram Sohrabi, Leili Pourafkari, Neda Roshanravan, Naser Aslanabadi, Farima Najjarian, Sina Mashayekhi, Samad Ghaffari

**Affiliations:** ^1^Cardiovascular Research Center, Tabriz University of Medical Sciences, Tabriz, Iran; ^2^Catholic Health System, Sisters of Charity Hospital, University at Buffalo, NY, USA

**Keywords:** Acute Coronary Syndrome, Neutrophil, Lymphocyte, NLR, Inflammation, Coronary Artery Diseases, SYNTAX Score, TIMI Risk Score, Myocardial Infarction

## Abstract

***Introduction:*** Considering the role of inflammation in pathogenesis of atherosclerosis, we aimed to investigate the association of presentation neutrophil to lymphocyte ratio (NLR) with complexity of coronary artery lesions determined by SYNTAX score in patients with non-ST-elevation acute coronary syndrome (NSTE-ACS).

***Methods:*** From March 2018 to March 2019, we recruited 202 consecutive patients, who were hospitalized for NSTE-ACS and had undergone percutaneous coronary intervention in our hospital. The association of presentation NLR with SYNTAX score was determined in univariate and multivariate linear regression analysis.

***Results:*** Higher NLR was significantly associated with higher SYNTAX score (beta = 0.162, *P* = 0.021). In addition, older age, having hypertension, higher TIMI score, and lower ejection fraction on echocardiographic examination were significantly associated with higher SYNTAX score. TIMI score had the largest beta coefficient among the studied variables (TIMI score beta = 0.302, *P* < 0.001). In two separate multivariate linear regression models, we assessed the unique contribution of NLR in predicting SYNTAX score in patients with NSTE-ACS. In the first model, NLR was significantly contributed to predicting SYNTAX score after adjustment for age, sex, and hypertension as covariates available on patient presentation (beta = 0.142, *P* = 0.040). In the second model, NLR was not an independent predictor of SYNTAX score after adjustment for TIMI score (beta = 0.121, *P* = 0.076).

***Conclusion:*** In NSTE-ACS, presentation NLR is associated with SYNTAX score. However, NLR does not contribute significantly to the prediction of SYNTAX score after adjustment for TIMI score. TIMI risk score might be a better predictor of the SYNTAX score in comparison to NLR.

## Introduction

Patients with the non-ST-elevation acute coronary syndrome (NSTE-ACS), constitutes a large proportion of patients with ACS.^1^ The management of patients with NSTE-ACS requires a thorough evaluation of patients’ prognosis by determination of validated risk scores.^2^ In addition, the complexity of coronary lesions affects the prognosis and revascularization methods in these patients.^2^ So, besides the risk evaluation scores, which are commonly determined on presentation, the SYNTAX score is commonly determined during coronary angiography to indicate the complexity of coronary lesions.^2^ However, predicting the magnitude of coronary involvement on presentation may yield important information for choosing the best therapeutic approach.

Considering the crucial role of inflammatory cells in the pathogenesis of atherosclerosis and acute coronary syndromes, neutrophil to lymphocyte ratio (NLR) has been recently introduced as a possible prognostic marker in patients with coronary heart disease.^3-7^ Furthermore, in the course of ACS, acute ischemic changes may cause changes in the number of circulating inflammatory cells including neutrophils and lymphocytes. As a result, investigating the potential role of NLR as a simple, inexpensive, and highly accessible prognostic factor in each separate subgroup of patients in the wide spectrum of coronary artery diseases is of great importance. However, there are only a few studies that have explored the role of NLR in patients with NSTE-ACS exclusively. On the other hand, the findings of the studies regarding the possible role of NLR in predicting the severity of coronary involvement in this patient population are controversial.^8,9^

Regarding these issues, in a prospectively recruited study in patients with NSTE-ACS who underwent coronary angiography, we evaluated the role of presentation NLR in predicting SYNTAX score.

## Materials and Methods

### Study population and design

In this prospective study, we enrolled 202 consecutive patients who were hospitalized for NSTE-ACS and had undergone percutaneous coronary intervention in our tertiary level cardiovascular hospital from March 2018 to March 2019. Patients with hematologic diseases, autoimmune disorders, active or chronic infectious diseases, hepatic cirrhosis, severe valvular diseases, and those who were pregnant were excluded from this study. Research ethics committee of Tabriz University of Medical Sciences reviewed the study protocol and approved this study. Regarding the descriptive nature of the study, it was exempt from obtaining informed consent. However, complete patient privacy was protected in all steps of the study.

Demographic information, past medical history, laboratory data, echocardiographic and angiographic findings of patients were entered into prepared questionnaires. Thrombolysis in Myocardial Infarction (TIMI) score was calculated for each patient using the TIMI scoring system.^10^

The NLR of patients on presentation was classified into three tertiles and compared regarding the basic clinical information, echocardiographic and angiographic findings. The association of presentation NLR with subsequently calculated SYNTAX score in angiography was investigated in both univariate and multivariate linear regression analysis. The association of NLR with SYNTAX score was also investigated with adjustment for TIMI risk score which is also readily available on patient presentation.

### Angiographic examination

The standard femoral approach with a 7-Fr guiding catheter was employed for percutaneous coronary intervention. A certified cardiologist who was blinded to the patient information reevaluated the digital angiographic records before angioplasty and calculated the SYNTAX score for each patient. The SYNTAX score was calculated using the standard SYNTAX scoring algorithm.^11,12^

### Laboratory examination

On presentation of patients to the emergency room, the venous blood was collected in citrated tubes having potassium ethylenediaminetetraacetic acid as an anticoagulant. Automated and daily-calibrated Coulter CBCH1 counter was used by the hospital laboratory to determine total white blood cell count, neutrophil count, and lymphocyte count. NLR was defined as an absolute neutrophil count per mm^3^ of blood divided by the absolute lymphocyte count per mm^3^ of blood.

### Statistical analysis

Baseline characteristics are presented as frequency and percentage for categorical variables and mean with standard deviation or median with 25%-75% interquartile range for continuous variables. Comparison of categorical variables was made by chi-square test or Fischer’s exact test as appropriate. Comparison of continuous variables with normal distribution was made by independent student *t* test, or ANOVA test and comparison of continuous variables with non-normal distribution was made by Mann–Whitney U test or Kruskal–Wallis test as appropriate. The comparison of study variables was made between low, intermediate, and high NLR groups. The association of study variables with the SYNTAX score was investigated in univariate linear regression analysis. In two separate multivariate linear regression analyses, the association between NLR and SYNTAX score was determined after adjustment for covariates in linear regression analysis. The significant predictors of SYNTAX score in the univariate analysis which are available on patient presentation in addition to patient’s sex, which is shown to be influential on NLR in a healthy population^13^ were entered into multivariate analysis. Statistical significance was determined using a 2-sided alpha level of 0.05. IBM SPSS Statistics software version 22 was used for analyzing data.

## Results

### Patient characteristics

The mean age of the patient population was 62.0  ±  10.7 years. Among 202 patients 69.3% were male, and 30.7% were female. The mean NLR value in our sample was 3.44  ±  3.22 and the median (25%-75% interquartile range [IQR]) was 2.20 (1.50-3.60). Based on the NLR tertile, patients with NLR value of less than 1.7 was considered as low NLR group. Patients with NLR value of ≥1.7 to < 3 were considered as intermediate NLR value and patients with NLR value of ≥3 were considered as high NLR value. The mean SYNTAX score was 17.73  ±  10.16 and the median (25%-75% IQR) was 17 (9-25). In 86 patients (42.6%) involvement of three coronary arteries or left main coronary artery was present in the coronary angiographic examination. One-vessel involvement and two-vessel involvement was present in 49 patients (24.3%) and 67 patients (33.2%), respectively.

### Comparison of the patient sample regarding the NLR group

Table 1 presents the comparison of demographic information, coronary risk factors, echocardiographic, and angiographic data in three NLR groups. The mean age of patients of the three groups was similar. 54.8% of low NLR group, 69.0% of intermediate NLR group, and 82.6% of high NLR group were male, which was significantly different among groups (*P* = 0.003). The prevalence of hypertension, hyperlipidemia, and diabetes was similar in three groups. The prevalence of smoking was 19.4%, 35.2%, and 40.6% in low, intermediate, and high NLR groups, respectively (*P* = 0.027). The family history of cardiovascular diseases was present in 4.8% of the low NLR group and it was present in 19.7% of intermediate and 5.8% of high NLR groups (*P* = 0.006). Patients in high NLR group had significantly lower ejection fraction on presentation (41.5% ±  11.9% vs. 46.6%  ±  8.6% vs. 50.0%  ±  6.5% in high, intermediate and low NLR groups, respectively, *P* = 0.001).

**Table 1:** Cardiovascular risk factors, echocardiographic and angiographic findings in three tertiles of neutrophil to lymphocyte ratio

	**Neutrophil to Lymphocyte Ratio**			***P *** **value**
	**Low** **N: 62**	**Intermediate** **N:71**	**High** **N:69**	
Age (Years)	61.00 ± 10.56	61.45 ± 11.02	63.51 ± 10.58	0.354
Sex (Male)	34(54.8%)	49(69.0%)	57(82.6%)	0.003
Diabetes Mellitus	17(27.4%)	24(33.8%)	21(30.4%)	0.727
Hypertension	34(54.8%)	39(54.9%)	38(55.1%)	0.998
Hyperlipidemia	12(19.4%)	18(25.4%)	12(17.4%)	0.482
Smoking	12(19.4%)	25(35.2%)	28(40.6%)	0.027
Family History of CAD	3(4.8%)	14(19.7%)	4(5.8%)	0.006
TIMI Score	1.79 ± 1.26	2.04 ± 1.21	2.48 ± 1.43	0.010
Ejection Fraction (%)	50.00 ± 6.56	46.61 ± 8.68	41.55 ± 11.90	0.001
SYNTAX Score	14.86 ± 9.28	16.74 ± 10.16	21.34 ± 10.02	0.001
**Number of Involved Vessels**				
One-Vessel	19(30.6%)	20(28.2%)	10(14.5%)	
Two-Vessel	22(35.5%)	23(32.4%)	22(31.9%)	
Three vessel / LMCA	21(33.9%)	28(39.4%)	37(53.6%)	0.108
Hospital Mortality	0(0.0%)	1(1.6%)	2(3.0%)	0.407
Three vessel / LMCA	21(33.9%)	28(39.4%)	37(53.6%)	0.059
**Follow up Recommendation**				
Conservative Management	6 (9.7%)	7 (9.9%)	6 (8.7%)	
CABG	21 (33.9%)	31 (43.7%)	43 (62.3%)	
Staged PCI	35 (56.5%)	33 (46.5%)	20 (29.0%)	0.019

Abbreviation: CABG, coronary artery bypass grafting; CAD, coronary artery disease; LMCA, left main coronary artery disease; PCI, percutaneous coronary intervention

**Figure 1:**
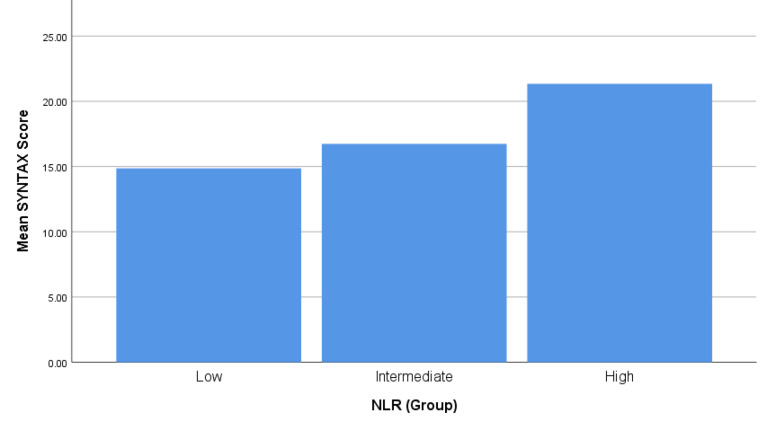
The mean SYNTAX score in low, intermediate and high neutrophil to lymphocyte levels

**Figure 2:**
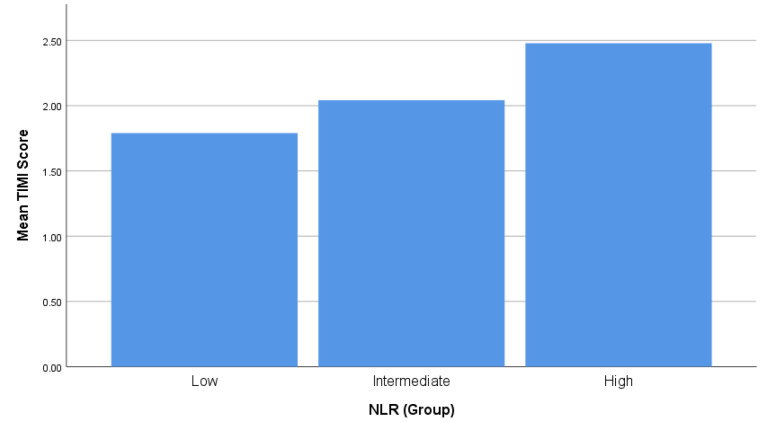
The mean TIMI score in low, intermediate and high neutrophil to lymphocyte levels

Figure 1 shows the mean SYNTAX score in three NLR groups. The mean SYNTAX score was significantly higher in the high NLR group. (SYNTAX score: 14.86  ±  9.28 vs. 16.74  ±  10.16 vs. 21.34  ±  10.02, *P* = 0.001). The results of post-hoc comparisons with the Tukey HSD test revealed that the mean SYNTAX score of high NLR group was significantly different from the intermediate NLR group (*P* = 0.017) and also from the low NLR group (*P* = 0.001). The mean SYNTAX score in the intermediate NLR group did not differ significantly from the low NLR group (*P* = 0.518). Figure 2 depicts the mean TIMI score in three NLR groups. Patients of higher NLR group had significantly higher TIMI scores. The mean TIMI score was 1.79  ±  1.26, 2.04  ±  1.21, and 2.48  ±  1.43 in low, intermediate and high NLR groups (*P* = 0.010). Prevalence of three-vessel disease or left main coronary artery stenosis was 33.9% in low NLR group, 39.4% in intermediate and 53.6%, in high NLR groups, *P* = 0.059. In addition, patients in higher NLR group were significantly more likely to be referred for coronary artery bypass grafting after undergoing angiography (62.3% vs. 43.7% vs. 33.9% in high, intermediate and low NLR groups, respectively, *P* = 0.019)

### The association of NLR with SYNTAX score

Table 2 presents the results of the univariate linear regression analysis of NLR and other covariates, with SYNTAX score in patients with NSTE-ACS. Higher NLR was significantly associated with higher SYNTAX score (beta = 0.162, *P* = 0.021). Also, older age, having hypertension, higher TIMI score, and lower ejection fraction on echocardiographic examination were significantly associated with higher SYNTAX scores. TIMI score had the largest beta coefficient among the studied variables (TIMI score beta = 0.302, *P* <  0.001).

**Table 2:** Univariate linear regression analysis between NLR and other covariates, and SYNTAX score

	**β (95% CI)**	***P *** **Value**
Age	0.24 (0.10-0.35)	0.001
Male sex	0.09 (-1.00- 5.10)	0.187
Diabetes mellitus	0.09 (-1.01- 5.08)	0.190
Hypertension	0.22 (1.77- 7.31)	0.001
Hyperlipidemia	0.10(-0.90- 6.02)	0.147
Smoking	-0.01 (-3.23- 2.81)	0.891
Family history	-0.08 (-7.37- 1.85)	0.240
TIMI score	0.30 (1.29- 3.33)	< 0.001
Neutrophil to lymphocyte ratio	0.16 (0.07- 0.94)	0.021
Highest NLR tertile	0.25 (2.59 - 8.35)	< 0.001
Left ventricular ejection fraction	-0.22 (-0.40- -0.08)	0.003

Abbreviation: NLR, neutrophil to lymphocyte ratio; TIMI,Thrombolysis in Myocardial Infarction; NLR, neutrophil to lymphocyte ratio, CI, confidence interval

In two separate multivariate linear regression models, we assessed the unique contribution of NLR in predicting SYNTAX score in patients with NSTE-ACS (Table 3). In the first model, NLR was significantly contributed to predicting SYNTAX score after adjustment for age, sex, and hypertension as covariates available on patient presentation (beta = 0.142, *P* = 0.040). R2 was 0.113 for the first model. In the second model, NLR did not contribute significantly to the prediction of SYNTAX score after adjustment for the TIMI score, which is also available on presentation (beta = 0.121, *P* = 0.076). R2 was 0.106 for model 2.

**Table 3:** Multiple linear regression analysis investigating independent association between neutrophil to lymphocyte ratio and SYNTAX score

	**β (95% CI)**	***P*** ** Value**
**Model 1**		
Age	0.20(0.06-0.32)	0.003
Male sex	0.12(-0.35-5.74)	0.083
Hypertension	0.21 (1.54-7.13)	0.003
Neutrophil to lymphocyte ratio	0.14 (0.02-0.87)	0.040
**Model 2**		
TIMI score	0.28 (1.15-3.20)	< 0.001
Neutrophil to lymphocyte ratio	0.1(-0.04-0.80)	0.076

Abbreviation: CI,confidence interval; TIMI,Thrombolysis in Myocardial Infarction Data are presented as regression coefficients (β) and 95% confidence interval. R2 is 0.113 for model 1 and R2 is 0.106 for model 2.

## Discussion

In this study, we found a positive association between presentation NLR and SYNTAX score in patients with NSTE-ACS. Higher NLR at presentation was an independent predictor of higher SYNTAX score after adjustment for age, sex, and hypertension as covariates. However, with adjustment for the TIMI score, NLR was not an independent predictor of the SYNTAX score.

In recent years, there is growing evidence regarding the role of neutrophils in the development of atherosclerotic plaque as well as destabilization and rupture of plaques.^14,15^ In addition, adaptive immunity including lymphocytes has a major role in the pathogenesis of atherosclerosis.^16^ Along with experimental studies, there is growing clinical evidence emphasizing the prognostic importance of changes of inflammatory cells in the setting of coronary artery diseases both in acute and chronic settings.^14,17^ Furthermore, the acute stress state and acute inflammatory reaction following the acute coronary diseases can lead to increased plasma cortisol, and consequently an increased number of circulating neutrophils along with a decreased number of circulating lymphocytes. This may contribute to the changes seen in early phases and may also differ based on the type of acute coronary syndrome. ^18-20^

As shown in our study, in patients with NSTE-ACS, presentation NLR was associated with angiographic SYNTAX score. SYNTAX score is an angiographic risk stratification score, used for quantifying the magnitude of coronary involvement and choosing the appropriate revascularization method based on the clinical context in each patient. In patients with NSTE-ACS, some studies have investigated the utility of presentation NLR for predicting the angiographic SYNTAX score with controversial results. Altun et al, found NLR as an independent predictor of high SYNTAX score group in patients with NSTE-ACS. In their study, NLR with a cut-off point of 3.46 had 67% sensitivity and 85% specificity for predicting high SYNTAX score group.^5^ In another study, Kurtul et al compared NSTEMI patients with low SYNTAX scores to patients with intermediate or high SYNTAX scores in univariate analysis and found lower NLR in the group with low SYNTAX score. Similar to our findings, they found NLR as an independent predictor of the SYNTAX score in linear regression analysis.^6^ Soyla et al studied three NLR tertiles in patients with NSTE-ACS. In accordance with the results of our study, they also found that patients in the high NLR group had higher SYNTAX score values in univariate analysis. However, they also investigated the correlation of SYNTAX score with NLR and found that a positive correlation was present only in patients of the high NLR group and not in the low or intermediate group.^21^ Zuin et al studied the association of NLR and SYNTAX score in patients with NSTEMI in a univariate analysis and found a higher SYNTAX score in patients of higher NLR tertile group. There was also a positive correlation between NLR and the SYNTAX score. Unlike our study, the association of NLR and SYNTAX score was not investigated in multivariate analysis.^22^

Our findings regarding the positive association of presentation NLR with SYNTAX score in patients with NSTE-ACS are in line with most studies. However, regarding the beta coefficient of 0.162 in univariate analysis and the beta coefficient of 0.142 in multivariate analysis, this can be considered as a weak association. Moreover, for the first time, we investigated the clinical utility of NLR for prediction of the SYNTAX score in NSTE-ACS by considering TIMI score as a covariate, which is also commonly determined on patient presentation and found TIMI score but not NLR as an independent predictor for SYNTAX score. TIMI risk score is a validated system for predicting the prognosis of patients with NSTE-ACS^10^ and it is a useful method for determining the appropriate therapeutic approach in this patient population.^2^ Higher TIMI risk score has been also suggested as a predictor of the severity of coronary artery disease in patients with NSTE-ACS.^23^ However, the association of TIMI score with SYNTAX score is not well investigated. Unlike our findings, in a study by Korkmaz et al, the TIMI risk score was not associated with SYNTAX score in patients with NSTE-ACS.^24^ In contrast, Hammami et al found a positive moderate correlation between the TIMI score and the SYNTAX score. However, the TIMI score was not able to predict the severe SYNTAX score.^25^ While the TIMI risk score is not currently validated for prediction of SYNTAX score, we found TIMI risk score a better predictor of the SYNTAX score in comparison to NLR.

There are some limitations to this study that should be mentioned. Our study is a single-center observational research with limited sample size. In addition, NLR was measured only on patient presentation, and information about the serial changes of NLR value was not available. While patients with various diseases that might affect the inflammatory markers were excluded from this study, there was the possibility of the presence of other unknown inflammatory conditions that might influence the results. We only included patients who presented to our tertiary cardiovascular center with NSTE-ACS and did not have access to the information regarding the out-of-hospital deaths due to NSTE-ACS.

## Conclusion

Our findings suggest that higher presentation NLR is associated with higher SYNTAX score in patients with NSTE-ACS. While higher NLR is found to be independently associated with SYNTAX score after adjustment for age, sex, and hypertension in multivariate analysis, considering TIMI risk score as a covariate in the model, NLR is not an independent predictor of SYNTAX score. TIMI risk score might be a better predictor of the SYNTAX score in comparison to NLR.

## Acknowledgements

Not applicable.

## Competing interest

The authors declare that they have no competing interests.

## Ethical approval

Research ethics committee of Tabriz University of Medical Sciences reviewed and approved this study. All procedures were in accordance with the ethical standards of the responsible committee on human experimentation of Tabriz University of Medical Sciences and with the Helsinki Declaration of 1975, as revised in 2013.

## Funding

None.
